# A perinatal review of singleton stillbirths in an Australian metropolitan tertiary centre

**DOI:** 10.1371/journal.pone.0171829

**Published:** 2017-02-13

**Authors:** Supuni Kapurubandara, Sarah J. Melov, Evangeline R. Shalou, Monika Mukerji, Stephen Yim, Ujvala Rao, Zain Battikhi, Nirusha Karunaratne, Roshini Nayyar, Thushari I. Alahakoon

**Affiliations:** 1 Westmead Institute for Maternal and Fetal Medicine, Westmead Hospital, Sydney, New South Wales, Australia; 2 University of Sydney, Sydney, New South Wales, Australia; 3 Liverpool Hospital, Sydney, New South Wales, Australia; 4 Nepean Hospital, Sydney, New South Wales, Australia; BC Children's Hospital, CANADA

## Abstract

It is estimated that everyday 7000 women worldwide have their pregnancy end with a stillbirth, however, research and data collection on stillbirth remains underfunded. This stillbirth case series audit investigates an apparent rise in stillbirths at a Sydney tertiary referral hospital in Australia. A retrospective case series of singleton stillbirths from 2005–2010 was conducted at Westmead Hospital. Stillbirth was defined as per the Perinatal Society of Australia and New Zealand classification as a death of a baby before or during birth, from the 20th week of pregnancy onwards, or a birth weight of 400 grams or more if gestational age is unknown. A total of 215 singleton stillbirths were identified in a cohort of 28 109, a rate of 7.6 per 1000 singleton births. There was a significant increase in annual stillbirth rate at our institution; the rate exceeded both Australian national and state singleton stillbirth rates. After pregnancy terminations over 20 weeks were excluded from the data, there was no statistical change in the stillbirth rate over time. Congenital anomalies (27%) and unexplained antepartum death (15%) remained as major causes; fetal growth restriction (17%) was also identified as an increasingly important cause, particularly in preterm gestations. Termination of pregnancy after 20 weeks was found to be the cause of rising stillbirth rate at our institution. Local and national data collection on stillbirth should be standardised and should include differentiation of termination of pregnancy as a separate entity so as to accurately assess stillbirth to target appropriate research and resource allocation.

## Introduction

There are more than 2.6 million stillbirths worldwide every year beyond 28 weeks gestation, with estimates of over 7000 women experiencing stillbirth every day. [1–3] The majority (98%) of stillbirths occur in low and middle income nations, many of which are under-reported. [4] However this significant burden on families has improved from estimates of 24.7 per 1000 births in the year 2000 to currently 18.4 per 1000 births, [1] which has remained stable over the last five years. [5] Stillbirth is still the largest contributor to perinatal death rate and is ten times more common than sudden and unexpected infant deaths. [6]

Overall there has been a decline in the rate of stillbirths in high income countries, however Australia has not reflected these trends. [2, 7, 8] In 2013 there were 2 191 stillbirths in Australia, a stillbirth rate of 7 per 1000, [9] stable from a stillbirth rate of 6.7 per 1000 births in 2002. [10]

The cause of majority of stillbirths in high income countries remain unexplained, accounting for 27–75% of all cases, varying according to the classification system used. [10, 11] Congenital anomaly has been documented as the leading cause of known stillbirths in Australia. [12, 13] The Australian Institute of Health and Welfare (AIHW) 2013 [9] report found a rising rate of stillbirths amongst the 20–23 week gestational age group in Australia, stating that terminations were unable to be distinguished from all stillbirths in this subgroup.

Despite the high prevalence of stillbirth globally, the UN Millennium Development Goals did not address stillbirth as a significant public health concern. This significant omission to an essential public health tragedy is readdressed in the 2014 Every Newborn Action Plan (ENAP) that is coordinated by UNICEF and the World Health Organisation (WHO) and aimed at reducing preventable newborn and fetal deaths. [14] ENAP sets the difficult world goal of a stillbirth rate of 10 per 1000 by 2035. [15] Despite the prominence placed to this significant global issue, there remains a lack of substantial research in this area. [16, 17] A recent review for an effective global stillbirth and neonatal classification system, documented and compared 81 different systems used with no method satisfying more than 7 of 17 expert-determined domains for effective classification. [18] The lack of adequate standardised methods of investigating, collecting and reporting data is critical for the prevention, management and understanding of stillbirth. [19–21] Perinatal Society of Australia and New Zealand (PSANZ) stillbirth clinical practice guideline was developed in order to standardise the investigations, data collection, classifications and reporting of perinatal deaths and to enable analysis of such data but needs effective implementation. [22] PSANZ Perinatal Death Classification (PSANZ-PDC) defines stillbirth as the death of a baby before or during birth, from the 20th week of pregnancy onwards, or a birth weight of 400 grams or more if gestational age is unknown. [23] Across Australia there is inconsistent reporting on termination of pregnancy and exclusion or inclusion of terminations from stillbirth records. Hence it has not been possible to reliably separate terminations from other causes of stillbirth in Australia. [12]

In 2013 Westmead Hospital, a major tertiary teaching hospital had a catchment population of 876 500 with approximately 43% being born overseas, compared to only 27% across the state of New South Wales. [24] The literature suggests that ethnicity and country of birth are risk factors for stillbirth. [25–27]

The primary aim of the study was to review the incidence, causes of singleton stillbirth and any recent trends at our institution in the context of a demographically diverse obstetric population over a six-year period.

## Materials and methods

A retrospective case series review was conducted using data extracted from the hospital obstetric database. All singleton stillbirths over a six-year period from January 1^st^ 2005 to December 31^st^ 2010 at our institution were included in the study. Multiple pregnancies were excluded from this analysis. Local and regional designated referral pathways based on place of residence were consistent during the study period.

The review was conducted in accordance to PSANZ perinatal mortality audit guidelines. The standard pro forma was completed individually for each stillbirth after careful review of hospital records. The pro forma was modified to incorporate consanguinity, which was relevant to the study population.

A total of 235 singleton stillbirths were identified. Subsequently 20 were omitted from analysis, due to missing medical records (*n = 4*), incorrect classification of stillbirth (*n = 6*) and patient transferred to our institution following stillbirth occurring at another hospital prior to transfer (*n = 10*).

A team of specialists in maternal fetal medicine analysed 215 files that were available for review to ascertain the cause of stillbirth according to the PSANZ Perinatal Death Classification system (PSANZ-PDC). Fetal growth restriction was defined as per the PSANZ-PDC category 8, inclusive of birth weight of <10th percentile of gestational age in a non-macerated stillbirth and or confirmed diagnosis of growth restriction by antenatal ultrasound. [23]

Data collected and used for analysis included primary cause of stillbirth, gestational age, maternal demographics, antenatal complications and postpartum maternal outcomes. Investigations for stillbirth and follow-up data according to PSANZ perinatal guidelines were also collected.

The statistical software package SPSS, version 21 (SPSS Inc. Chicago Illinois) was used to analyse the data collected. Cross-tabulation between stillbirth and different covariates was performed in order to define the characteristics of the study population. Descriptive analysis of stillbirth and antenatal care received, stillbirth investigations and postpartum follow up were also performed. Univariate logistic regression analysis was used to calculate the odds ratio (OR) and the 95% confidence interval to determine the trend in stillbirth rate over the study period. Overall cause of stillbirth and analysis of cause stratified by type of stillbirth (antepartum or intrapartum) and three gestational ranges (term ≥37 weeks, preterm 28–36^+6^ weeks and early preterm 20–27^+6^ weeks) were also performed.

Approval for the study was obtained from the Western Sydney Local Health District Human Research Ethics Committee.

## Results

During the study period, 2005–2010, 28 109 singleton deliveries were recorded at our hospital, this comprised 215 stillbirths for analysis. The overall singleton stillbirth rate varied from 6.2 to 9.4 per thousand singleton births over the study period (Table 1), with an overall rate of 7.6/1000 births. We noted a statistically significant rising trend in the singleton stillbirth rate (OR per year = 1.08, 95%CI 1.001 to 1.171, p = 0.048). However, after excluding termination of pregnancy (TOP *n* = 40) this was not significant (OR per year = 1.03, 95%CI 0.945 to 1.124, p = 0.49).

**Table 1:** Stillbirth statistics in Australia and Westmead Hospital 2005–2010.

Year	Australian Births	National SBR*	National singleton SBR*	Westmead Births	Westmead singleton SBR *	Westmead singleton SBR *#
2005	272 419	7.3	6.9	4 504	6.2	5.3
2006	282 169	7.4	7.0	4 520	7.1	6.6
2007	294 205	7.4	7.0	4 401	6.4	5.5
2008	296 925	7.4	6.9	4 688	7.7	6.4
2009	299 220	7.5	7.4	4 790	9.4	6.5
2010	299 563	7.4	7.0	5 206	8.6	6.5

National stillbirth rate without TOP is not available

* Per 1,000 Singleton Births

^#^ without TOP

In our study group of 215 mothers, 90 were Australian born and 125 were overseas born. The maternal ethnicity data was not available for the general obstetric population. Demographic data from the stillbirth population are presented in Table 2.

**Table 2:** Demographics and clinical characteristics of women with stillbirths.

Characteristics	Number of Stillbirths	Percentage of Stillbirths (%)
Maternal Age		
Age < 20	184	85.58
20–34	26	12.09
> 34	5	2.33
Parity		
Primipara	201	93.49
1–3	12	5.58
> 3	2	0.93
Gestational Age		
Early (20–27 weeks)	115	53.49
Preterm 28–36	59	27.44
Term (37–41)	41	19.07
Body Mass Index		
<18.5 kg/m^2^ (Underweight)	6	2.79
18.5–24.9 kg/m^2^ (Normal)	64	29.27
25–29.9 kg/m^2^ (Overweight)	49	22.79
≥ 30 kg/m^2^ (Obese)	40	18.40
Unknown	56	26.05
Smoker		
Yes	28	13.02
No	181	84.19
Unknown	6	2.79
Alcohol Intake		
Yes	5	2.33
No	205	95.35
Unknown	5	2.33
Illicit Drug Use		
Yes	5	2.33
No	205	95.35
Unknown	5	2.33
Consanguinity		
Yes	28	13.02
No	156	72.56
Unknown	31	14.42
Artificial Reproductive Technology		
Yes	9	4.19
Clomiphene	6	2.79
IVF	3	1.40
No	205	95.35
Unknown	1	0.47

A majority of women (84%) in this study who had a stillbirth were non-smokers and we were not able to assess smoking as a risk factor for stillbirth.

**Fig 1:**
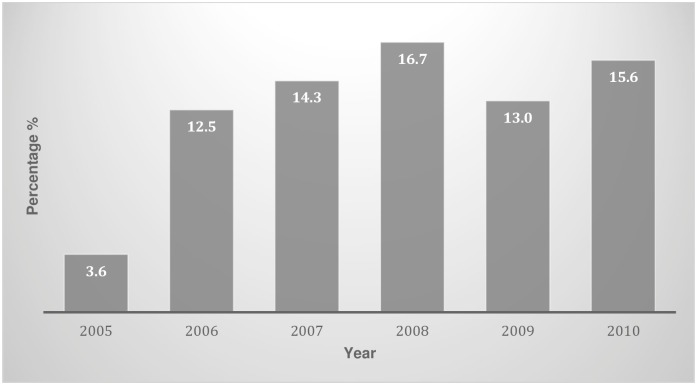
Percentage of consanguinity in Westmead stillbirth population from 2005–2010.

Parental consanguinity was a feature in 28 of 184 stillbirths (15%), with 31 (14%) cases missing this data. Between 2005 and 2010, consanguinity rate amongst those who had a stillbirth increased (Fig 1).

Thirty seven percent of the study group reported decreased fetal movement in the week preceding the stillbirth. Within this sub group, the predominant causes of stillbirth was due to unexplained antepartum death (27%) followed by fetal growth restriction (22%) and congenital anomalies (20%). Five percent of women had not booked for antenatal care prior to their diagnosis of stillbirth.

**Fig 2:**
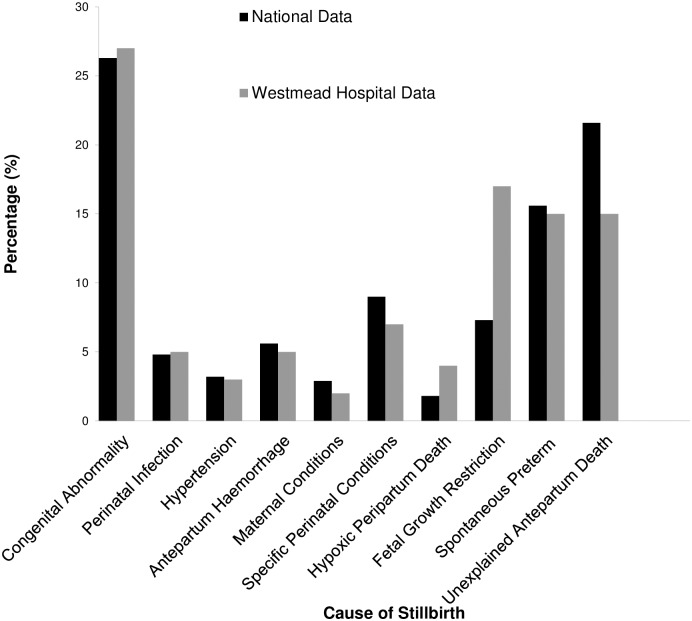
Cause of stillbirth using PSANZ perinatal death classification- comparison of Westmead Hospital singleton data and national data.

The most common causes of singleton stillbirth were congenital abnormality (27.4%), intrauterine growth restriction (IUGR; 16.7%), threatened preterm labour (14.9%) and unexplained stillbirth (14.9%). The majority of the stillbirths (78%) occurred in the antepartum period (Fig 2). There were a total of 46 stillbirths classified as intrapartum death. Of these 15 were due to termination of pregnancy, 8 were at gestations of extreme prematurity (<24/40) and 7 were due to congenital anomalies as a cause of death. This results in an intrapartum stillbirth rate of 9% (16/175) which included 3 cases of stillbirth classified as hypoxic peripartum death.

The causes of stillbirth varied according to gestational age. At term, the most common cause of stillbirth was unexplained antepartum death (39%), perinatal infections (13.9%) and congenital abnormalities (13.9%). In preterm gestations (28–36^+6^ weeks), congenital abnormalities (32.8%), intra uterine growth restriction (17.2%) and antepartum haemorrhage (15.5%) were the most common causes. In early preterm gestations (20–27^+6^ weeks) congenital abnormality (29.3%), preterm labour (26.7%) and IUGR (19.8%) were the top three causes of stillbirth. Disorders such as hypertension are classified as per criteria set in PSANZ-PDC section 7 (https://www.psanz.com.au/assets/Uploads/Section-7-Version-2.2-April-2009.pdf) with expert consensus that the condition was the primary issue that resulted in stillbirth.

Uptake of stillbirth investigations was analysed after the exclusion of terminations of pregnancy (*n* = 40). Although postmortem was offered in 87% of the cases, the uptake was only 42%. Of the unexplained antepartum stillbirths, 53% had a postmortem performed.

Complete maternal blood testing, as recommended by PSANZ guidelines (Perinatal Society of Australia and New Zealand, 2009 https://www.psanz.com.au/assets/Uploads/Section-5-Version-2.2-April-2009.pdf), was undertaken in 50% of women with stillbirth and partial tests in 38%. Other investigations included ultrasound prior to delivery (45%), placental histopathology (93%) and amniocentesis (5%). There were 159 placental histopathology results available, excluding the 33 pregnancy terminations reports. Only 17% (*n* = 27) of placental histopathology results were reported as normal; 30% (*n* = 47) showed evidence of placental insufficiency, 33% (*n* = 52) showed changes associated with infection while 16% (*n* = 26) had pathology associated with the umbilical cord.

The reason given for termination of pregnancy (*n* = 40) were varied with, 40% (*n* = 16) were for fetal anomaly, genetic cause 28% (*n* = 11), 4 terminations were for maternal indication (hypertension and preeclampsia), some of these conditions were considered fatal and no terminations were for social reasons.

Medical records documented medical follow-up in 64%, midwifery follow-up in 85% and bereavement counselling in 85% after discharge.

## Discussion

The data presented from our review demonstrated a rising annual singleton stillbirth rate, a trend beyond the Australian national rate between 2005 and 2010 (Table 1).

However, the stillbirth rate was found to be stable after terminations were identified and excluded from the cohort. Varying inclusion of termination of pregnancy in stillbirth figures throughout Australia and internationally provides a challenge to interpretation of stillbirth figures.

Bythell et al. [28] found there was a consistent overestimation of the real stillbirth rate due to termination of pregnancy. A Canadian study also revealed the need to have clear data as the rising stillbirth rate in their study group was the result of an increase in late termination of pregnancy. [29] The rising trend in Australian national stillbirth rate at an earlier gestation [12] may reflect an increase in termination as was uncovered at our institution. A large linked data study in Western Australia [30] from 1986–2010, found an increase in late termination of pregnancy had significant influence on stillbirth rates, concluding that disaggregated data was desirable for accurate analysis of trends in perinatal statistics. Flenady et al. [19] also called for standardised stillbirth data parameters, inclusive of details of termination to ease comparative studies between countries.

In the national data, 71.9% of confinements in 2010 were to Australian born mothers. [13] This is a significant contrast to our obstetric population with 41% born in Australia. Ethnicity and in particular the Indian subcontinent, has been identified to be associated with an increase in stillbirth rates [25–27], the contribution of this factor to stillbirth in our cohort is unknown and warrants further investigation.

Ethnicity is not recorded in the obstetric database and country of birth was the only relevant parameter available for our study. The literature suggests that recording ethnicity along with country of birth is needed for accurate appraisal of pregnancy outcomes in Australia and should be incorporated into obstetric/midwifery data collection. Further study into genetic differences and susceptibility to pregnancy complications as well as relevant cultural factors should be investigated to understand the risk of stillbirth in a multicultural obstetric population. We have identified consanguinity as a potential important risk factor for stillbirth in our population. Consanguinity as a risk factor for stillbirth has been suggested in other studies. [31–34] A previous study at this hospital during the same time period, revealed an overall consanguinity rate of 5.5%, less than the 15% found in this stillbirth case series. [34] Further investigation with a larger cohort is warranted into the clinical implications of consanguinity for stillbirth.

It is of interest that 5% of women in our audit were unbooked (i.e. had not booked-in hospital for antenatal care prior to presentation with stillbirth). Women that are unbooked and present late for antenatal care are known to be at greater risk for adverse fetal outcome. [34–36]

Our tertiary centre has a state-wide role in managing and delivering babies with surgical and congenital abnormalities due to its proximity to a large tertiary Children’s Hospital. The disparity between our institution and national stillbirth rates was not accounted for by this factor alone. The study demonstrated that stillbirth due to congenital abnormalities accounted for 27% of all cases, which was only marginally different to the national rate of 25.8% in 2010. [13] Interestingly, there was a difference in the rates of IUGR between the national and our hospital data. IUGR has emerged as one of the leading causes of singleton stillbirth in our hospital over this period accounting for 16.7% of stillbirths which is higher than the national rate of 8.8% in 2010. [13] Further study into ethnicity and causes of stillbirth such as IUGR may be beneficial to explore the higher rate of stillbirth associated with IUGR in our multicultural population. Growth restriction as a cause of stillbirth accounted for 20% of early preterm (<28 weeks), 17% of preterm (28–36^+6^ weeks) and 5.6% of term stillbirths (≥37 weeks). These results highlight the necessity to improve antenatal screening, detection and surveillance of IUGR, particularly in the preterm gestations. Recent publications have demonstrated that detection of IUGR can be accomplished by improved antenatal surveillance. [37, 38] This is of particular significance to our antenatal care framework, as low risk women are often not seen between 20 and 28 weeks of pregnancy.

Unexplained antepartum stillbirth remained in the top three causes of stillbirth despite 42% postmortem examinations and 93% having placental histological examinations (termination of pregnancy excluded). This study found that extra information was gained in 77% of cases through placental histopathogy uptake. [13] However of the 32 cases where no cause of death was found 31 had placental histopathology completed.

During this study universal placental examination was recommended but there was poor compliance with recommendations. Since this case review placental examination process at this hospital has improved, guidelines are adhered to and valuable information that can be gained from placental review are now more readily available. Postmortem examination is considered gold standard for stillbirth investigation as a cause might be found in 20–86% of all stillbirths. [39, 40] The postmortem rate is marginally higher than the state reported rate of 37.2%. This study also highlighted areas where clinical management can be improved with respect to completing all recommended investigations for stillbirth according to the PSANZ guidelines.

The importance of ongoing and persistent education with respect to the significance of fetal movement amongst pregnant women and obstetric staff was highlighted in this study by the fact that 37% of stillbirths had decreased movements in the week preceding adverse outcome. Additionally, this emphasises the need for clear protocols for management of decreased fetal movements. Set protocols for management of decreased fetal movements based on NSW health policy guideline, have been adopted at our institution since the study was undertaken.

Limitations of this study can be attributed to the retrospective methodology, which may lead to reporting and data collection bias. Recall bias may have effected some women who reported reduced fetal movement preceding stillbirth, due to the retrospective nature of the study it is difficult to delineate women who presented for assessment due to reduced fetal movement and those that may have been given leading questions on fetal movement after presenting for another reason. Consanguinity data collection on extent of relatedness is poor, with related or not related the only definition. Well-designed prospective studies, such as the Auckland Stillbirth study, would improve data quality to investigate the risk factors of stillbirth. [41] Variations in data collection throughout Australia and proven quality control of the data limits comparisons.

## Conclusion

In high income countries stillbirth still remains an ongoing tragedy for women and their families with little or no reduction in overall rate. Our study confirms that termination of pregnancy has to be clearly delineated from stillbirth data. Appropriate resources can then be allocated to researching unexplained stillbirth and any modifiable risk to reduce stillbirth such as fetal growth restriction. In the setting of a changing multicultural population, with increasing patient numbers and limited resources, prospective data collection and review will assist in improving clinical outcomes. This will enable institutions to review allocation of resources, clinical practice, improve guidelines, identify areas for research and ultimately reduce the rate of stillbirth.

Following this study our institution has implemented a formal perinatal review process of all stillbirths and a standardised stillbirth care plan. However further prospective and ongoing research and analysis is crucial to recognise the variables and trends associated with stillbirths to manage and prevent adverse perinatal outcomes in our population.
